# Improving the Well-Being of People With Advanced Cancer and Their Family Caregivers: Protocol for an Effectiveness-Implementation Trial of a Dyadic Digital Health Intervention (FOCUSau)

**DOI:** 10.2196/55252

**Published:** 2024-08-13

**Authors:** Peter Hudson, Jill Francis, Joachim Cohen, Suzanne Kapp, Richard De Abreu Lourenco, Lisa Beatty, Kathleen Gray, Michael Jefford, Ilona Juraskova, Laurel Northouse, Aline de Vleminck, Sungwon Chang, Patsy Yates, Sophy Athan, Shaira Baptista, Marlena Klaic, Jennifer Philip

**Affiliations:** 1 Centre for Palliative Care c/o St Vincent’s Hospital and The University of Melbourne Melbourne Australia; 2 End of Life Care Research Group Vrije Universiteit Brussel Brussels Belgium; 3 Melbourne School of Health Sciences The University of Melbourne Melbourne Australia; 4 Sir Peter MacCallum Department of Oncology The University of Melbourne Melbourne Australia; 5 Centre for Implementation Research Ottawa Hospital Research Ottawa, ON Canada; 6 Centre for Health Economics Research and Evaluation (CHERE) University of Technology Sydney Sydney Australia; 7 College of Education, Psychology and Social Work Flinders Institute for Mental Health and Wellbeing Flinders University Adelaide Australia; 8 Centre for Digital Transformation of Health The University of Melbourne Melbourne Australia; 9 Department of Health Services Research Peter MacCallum Cancer Centre Melbourne Australia; 10 Faculty of Science School of Psychology University of Sydney Sydney Australia; 11 School of Nursing University of Michigan Ann Arbor, MI United States; 12 Faculty of Health Improving Palliative, Aged and Chronic Care through Clinical Research and Translation (IMPACCT) University of Technology Sydney Sydney Australia; 13 Cancer and Palliative Care Outcomes Centre Queensland University of Technology Queensland Australia; 14 Cancer Consumer Advisory Committee Victorian Comprehensive Cancer Centre Alliance Melbourne Australia; 15 School of Public Health and Preventative Medicine Monash University Melbourne Australia; 16 St. Vincent’s Hospital and the Victorian Comprehensive Cancer Centre The University of Melbourne Melbourne Australia

**Keywords:** advanced cancer, clinical trial, digital health intervention, palliative care, health economics, implementation science, wellbeing, well-being, cancer, family caregiver, family caregivers, caregivers, caregiver, digital health, quality of life, dyad, self-administered, web-based intervention, web-based, Australian, Australia, efficacy, cost-effectiveness, psychoeducation

## Abstract

**Background:**

Advanced cancer significantly impacts patients’ and family caregivers’ quality of life. When patients and caregivers are supported concurrently as a dyad, the well-being of each person is optimized. Family, Outlook, Communication, Uncertainty, Symptom management (FOCUS) is a dyadic, psychoeducational intervention developed in the United States, shown to improve the well-being and quality of life of patients with advanced cancer and their primary caregivers. Originally, a nurse-delivered in-person intervention, FOCUS has been adapted into a self-administered web-based intervention for European delivery.

**Objective:**

The aims of this study are to (1) adapt FOCUS to the Australian context (FOCUSau); (2) evaluate the effectiveness of FOCUSau in improving the emotional well-being and self-efficacy of patients with advanced cancer and their primary caregiver relative to usual care control group; (3) compare health care use between the intervention and control groups; and (4) assess the acceptability, feasibility, and scalability of FOCUSau in order to inform future maintainable implementation of the intervention within the Australian health care system.

**Methods:**

FOCUS will be adapted prior to trial commencement, using an iterative stakeholder feedback process to create FOCUSau. To examine the efficacy and cost-effectiveness of FOCUSau and assess its acceptability, feasibility, and scalability, we will undertake a hybrid type 1 implementation study consisting of a phase 3 (clinical effectiveness) trial along with an observational implementation study. Participants will include patients with cancer who are older than 18 years, able to access the internet, and able to identify a primary support person or caregiver who can also be approached for participation. The sample size consists of 173 dyads in each arm (ie, 346 dyads in total). Patient-caregiver dyad data will be collected at 3 time points—baseline (T_0_) completed prerandomization; first follow-up (T_1_; N=346) at 12 weeks post baseline; and second follow-up (T_2_) at 24 weeks post baseline.

**Results:**

The study was funded in March 2022. Recruitment commenced in July 2024.

**Conclusions:**

If shown to be effective, this intervention will improve the well-being of patients with advanced cancer and their family caregivers, regardless of their location or current level of health care support.

**Trial Registration:**

ClinicalTrials.gov NCT06082128; https://clinicaltrials.gov/study/NCT06082128

**International Registered Report Identifier (IRRID):**

PRR1-10.2196/55252

## Introduction

### Overview

Advanced cancer is a “family affair” significantly affecting the well-being of the person with cancer and their family [1]. Family caregivers are at the core of patient care; they are typically unpaid laypeople, comprising close family members or friends or others who provide a significant level of support (practical, social, or emotional) to the person with cancer. Additionally, their contributions alleviate some of the burden on the Australian health care system, resulting in significant cost savings [2]. As a result, addressing the needs of both patients and their family caregivers is a priority in many countries, with established standards, policies, and guidelines [3-5].

Furthermore, the World Health Organization advocates that palliative care should be “family centered,” enhancing the quality of life (QoL) of the patient and their family caregivers [6], and includes supporting those still receiving curative treatment in addition to those receiving end-of-life (EoL) care [7]. Palliative care, when effectively delivered, can restore choice regarding options for care [8], improve patients’ and caregivers’ sense of control, self-efficacy, coping ability, communication about the illness, and reduce emotional distress [9]. Research has shown the benefits of early access to palliative care for patients, their family caregivers, and the health care system; there has been an increased and proactive approach to early integration of palliative care to improve QoL and symptom management of patients and family caregivers [7]. Hence, intervening earlier in the disease trajectory to assist the family unit in preparing for and responding to the implications of life-threatening illness constitutes best practice.

Despite the policy, clinical and research evidence advocating a family approach to palliative care, systemic inadequacies such as the inability of many family caregivers to access health professional support and fragmented, inconsistent, variable palliative care services, along with the lack of preparation for death have impeded family-centered care [10]. Patients may undergo potentially unnecessary treatment, report high levels of pain, difficulty coping, poor physical and emotional health, and their EoL wishes not always upheld [10]. This translates to many caregivers reporting high emotional distress, unmet needs, and difficulties with providing complex home-based care, especially during the advanced stages of illness and EoL [11]. Around 40% of caregivers are reported to experience psychological distress [12] which is typically underrecognized [13]. Furthermore; these impacts may be more pronounced in underserved groups.

The overwhelming majority of Australians would prefer to die at home [14], yet Australian acute hospitals provide EoL care to approximately 50% of people who die [15]. Comprehensive family support is a key factor in determining whether the preference for home care and death can be realized [16]. Yet, effective systematically applied psychosocial support for family caregivers in the EoL context is still underdeveloped [16,17].

Patients and family caregivers experience illness together, with 1 person’s ability to cope affecting the other’s [18]. In a longitudinal study of dyads including people with advanced cancer and their caregivers, caregiver mental health at baseline significantly influenced patient mental health 3 months later [11], while in a study of dyads affected by brain cancer, greater caregiver competence in the caregiver role predicted longer patient survival [19]. Dyadic interventions, targeting the patient and family caregiver together, are more likely to result in better outcomes for both parties than single-target interventions and may be more cost-effective [18,20,21]. Consequently, dyadic interventions focusing on the QoL of both the patient and caregiver from the point of advanced disease diagnosis are necessary to promote well-being and cost-effective care.

Historically, much of the psychological and psychoeducational palliative care intervention research has been delivered in person, with demonstrated improvements in patient QoL [22] and family caregiver well-being, sense of preparedness, reduction of unmet needs, and more favorable bereavement outcomes [21,23-25]. While these in-person delivered interventions produce promising results, they can be time and resource-intensive. These resource requirements constitute a major barrier to longer-term implementation and integration of these interventions into the health care system, particularly in the context of current shortages of staff and other resources.

Digital health interventions offer an innovative modality for the delivery of health care services [26]. They provide considerable resource advantages to in-person interventions [27] such as improving, information-sharing, decision-making, and communication [26,28,29]. Advantages for users also include lower cost of delivery, greater reach (including for rural and remote areas), convenience, less travel time, reduced risk of infections due to reduced exposure associated with face-to-face consultations, and recipients feeling more empowered [27,29]. A meta-analysis of internet therapy studies has provided strong support for the adoption of internet-based psychological interventions [30] and the increasing use of digital health has ushered in a new era of patient-centered cancer care that moves beyond the traditional in-person care model [31].

Digital health within the context of palliative care should not be considered a replacement for, but complementary to, in-person care in this con (which has numerous benefits) [29]. Indications are that digital health communication in this context may result in patients and caregivers receiving more reliable information; it can be more feasibly applied; and at organizational and societal levels, digital health may contribute to more efficient and equitable use of resources [29]. Furthermore, disseminating dyadic interventions via the internet and other technologies may be more scalable from a health systems perspective [18]. Reduced labor costs, as well as lower ongoing program charges, suggest that the longer-term cost-effectiveness of digital health interventions compares favorably with traditional in-person care [18]. However, compared to the rapid increase of digital health interventions in other areas of health care, there is limited application of digital health interventions within advanced cancer and palliative care settings. No rigorously tested interventions applied systematically in Australia have targeted the psychosocial well-being of the patient-caregiver dyad [26,29]. There is an urgent need, therefore, to develop evidence-based, dyadic digital health approaches that are meaningful, accessible, and maintainable for integration into the Australian health system.

### Focus Intervention Background

The Family, Outlook, Communication, Uncertainty, Symptom management (FOCUS) intervention has been developed in accordance with guidelines for complex interventions [32-36]. Co-designed with consumers, patients, and family caregivers, the intervention consists of five core components underpinning the FOCUS acronym and they are (1) supporting *Family* involvement, (2) supporting *Outlook* and meaning, (3) increasing *Coping* effectiveness, (4) reducing *Uncertainty*, and (5) *Symptom* management. The original in-person FOCUS program was developed in the United States as a nurse-delivered in-person intervention, being multicomponent and psychoeducational in its framework, and providing tailored informational and emotional support to patients with advanced cancer and their family caregivers [32]. The efficacy of this in-person FOCUS intervention was demonstrated in 3 randomized control trials and 2 pilot effectiveness studies conducted in the United States [32-35,37], with the patient-caregiver dyads reporting significantly improved QoL and well-being, less negative appraisal of illness and caregiving, reduced uncertainty and hopelessness, improved communication, and enhanced self-efficacy. To make the FOCUS (United States) intervention more accessible, the main component (family involvement) of this in-person intervention was then adapted into a tailored, digital or web-based format [38]. Significant intervention effects were found in a pre-post study on dyads’ QoL, emotional distress, perceived benefits of illness or caregiving, and caregivers’ self-efficacy [38].

The strong empirical base and use of the FOCUS (United States) intervention have been acknowledged by the European Union (EU) through successful grant funding awarded to test European-adapted versions of both programs (in-person [FOCUS+] and digital [iFOCUS] in a phase 3 trial, currently being conducted across 6 countries (England, Ireland, Belgium, Denmark, the Netherlands, and Italy) [39-41].

For this study, we aim to test the FOCUS EU digital version (iFOCUS) for the Australian setting (“FOCUSau”), given the need to reach regional and rural areas and address the cost-effectiveness and maintainability issues that arise for in-person interventions.

### Research Aim and Hypotheses

We seek to determine the effectiveness and maintainability of a digital health intervention (iFOCUS) aimed at improving the well-being of patients with advanced cancer and their primary family caregivers. Our objectives are to (1) adapt iFOCUS to the Australian context to create FOCUSau; (2) examine the effectiveness of FOCUSau in improving the well-being (primary outcomes are emotional well-being and self-efficacy) of patients with advanced cancer and their primary family caregiver, compared to the control group (usual care); (3) compare the types and costs of health services used by participants in the intervention and control group; and (4) assess the acceptability, feasibility, and scalability of FOCUSau in order to inform the maintainable implementation of the intervention within the Australian health care system.

Our hypotheses are that compared to the control group, patent-caregiver dyads in the intervention group (who receive the intervention in addition to usual care) will, at 12 weeks follow-up, report (1) higher levels of emotional well-being and self-efficacy (ie, confidence in managing illness; primary outcomes) and (2) higher QoL, less negative appraisal of illness or caregiving, better communication about the illness, and improved coping ability (secondary outcomes). In addition, FOCUSau will be cost-effective compared with the control group in terms of the incremental cost (change in health care use) per additional participant with a meaningful change in (1) emotional well-being and (2) self-efficacy.

## Methods

The study addresses the objectives in 3 stages.

### Stage 1: Adaptation of iFOCUS to the Australian Health Care Setting (Objective 1)

The adaptation of iFOCUS to create FOCUSau will involve appraisal of the intervention by consumer representatives and research team participants along with detailed feedback to inform modifications. The process will be underpinned by the ADAPT process [42] whereby we will also evaluate the quality, acceptability, appropriateness, comprehensibility, accessibility, and feasibility of the intervention before and after adaptation. We have concurrently reviewed the compatibility of the intervention software with Australian digital health infrastructure and ensured alignment with Australian standards for digital accessibility, security, privacy, and shareability of personal data, and capacity to interoperate with other systems.

Consumer representatives and research team participants (n=16) viewed the iFOCUS intervention and provided detailed feedback to inform the modifications. Participants also completed the Adapted Mobile Application Rating Scale [43] and the Theoretical Framework of Acceptability questionnaire [44] and commented on the quality, acceptability, appropriateness, comprehensibility, accessibility, and feasibility of the intervention before and after adaptation. We collected data using REDCap (Research Electronic Data Capture; Vanderbilt University) [45] and analyzed data using descriptive statistics, thematic analysis [46], and content analysis [47]. Data analysis led to suggestions for modifications to the intervention which were voted favorably by participants. The full method and results of the adaptation study will be reported elsewhere.

### Stage 2: Pragmatic Phase 3 Hybrid Effectiveness-Implementation Trial of FOCUSau (Objectives 2 and 3)

#### Design

We chose to conduct a Hybrid type 1 implementation study that included a Phase 3 effectiveness component and an observational implementation study [48]. This integrated research design includes digital health evaluation [49] to account for the interaction between conventional health effects and human-computer engagement [50]. This includes (1) randomization of participating dyads to the intervention versus standard care (control group), to examine FOCUSau effectiveness in relation to clinical outcomes and health service use, while simultaneously (2) exploring acceptability, feasibility, maintainability, and scalability of the intervention—that are recognized in both implementation science methods [48] and digital health evaluation methods [51]. Hybrid designs have a dual focus—a priori assessment of clinical effectiveness and implementation [48]. These pragmatic designs offer novel ways of testing intervention effectiveness and potential uptake and are recommended for multisite psychosocial-related interventions in cancer care [52].

#### Participants

Patient inclusion criteria are diagnosis of advanced cancer; older than 18 years of age; able to comprehend written or spoken English; no visual, hearing, or cognitive impairment that would preclude participation; can commit to research participation requirements (including data collection and completion of the FOCUSau intervention if randomized to that group); able to access the internet (on their own desktop computer, laptop computer, or tablet device); and able to identify a primary support person or caregiver, who is an unpaid individual (not necessarily a partner or family member) who is providing them with physical, social, or emotional support. The patient exclusion criterion is involvement in an advanced cancer nondrug trial that focuses on improving QoL. Family caregiver inclusion criteria are identified by the patient as their primary support person who is related to them biologically, legally, or emotionally, and is willing to accept this support role; aged older than 18 years; no visual, hearing, or cognitive impairment that would preclude participation; commits to research participation requirements; and is able to access the internet. Dyad inclusion criterion is the capacity to effectively use the internet (as determined through a short practical internet-based exercise as part of the screening and consent process).

#### The FOCUSau Intervention

##### Aim and Core Features

The overall aim is to enhance dyads’ emotional well-being and self-efficacy, addressed by 5 core components of the intervention [32]. The transactional model of stress and coping underpins the intervention [53].

##### Delivery Mode, Dose, and Timeline

**Figure 1:**
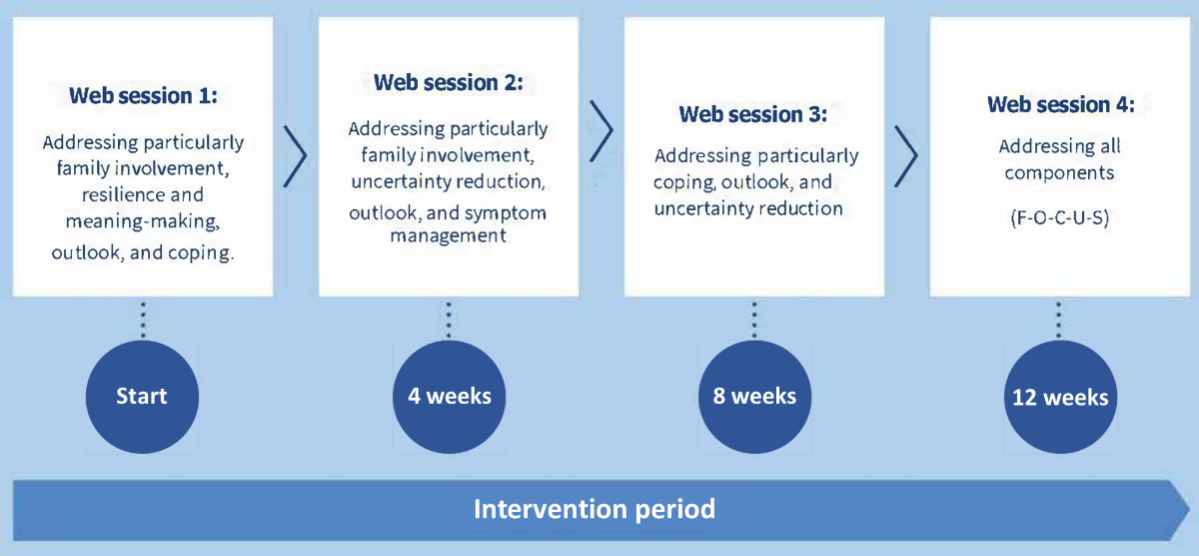
Intervention overview.

The intervention is self-administered, completed autonomously via the internet by the patient and caregiver dyads. It encompasses 4 prescribed consecutive sessions (with 3 weeks between each session) over a period of 12 weeks that collectively cover the 5 core components; the core content of which is outlined in Figure 1. The sessions are completed simultaneously by the patient and family caregiver, together at a computer. At the beginning of each session, a computer prompt (wizard) explains how dyads can navigate through the sessions. Access to the 4 sessions is provided via an automated link sent by an email. The content of the sessions is written at a lower secondary education reading level, using principles of plain language. Audio instead of text provision of material is also available to allow for participation by those with visual impairment. Written content is complemented by images and videos of patients and caregivers reflecting on their advanced cancer experience which is linked to 5 core components.

##### Additional Resources

Dyads are provided with an internet-based personal workbook containing information tailored to their responses to questions completed during the internet sessions. Any information sheets that the dyad indicated as “of interest to them” during the internet sessions are included as a hyperlink in their personal workbook which also contains evidence-based local advanced cancer-related resources. Assistance via a helpdesk (via email or telephone) is available to resolve any technical difficulties with accessing the internet-based materials.

##### Tailoring of the Intervention

Tailoring enhances the intervention’s efficacy by providing dyads with information personally relevant to them. Three types of tailoring strategies are used—personalization, tailored feedback, and content matching [54]. Participants receive tailored individual and dyadic messages according to their own demographic characteristics (age, sex, and dyadic relationship) provided at study enrollment and their responses to questions within the sessions.

#### The Control—Usual Care

Participants in the control group will receive usual care. Usual care in advanced cancer care is known to be heterogeneous. Nonetheless, we will collect relevant health services usage data (see below) to describe and compare the control and intervention arms.

#### Setting and Recruitment Procedures

The patient-caregiver dyads will be recruited via two methods that are (1) referral from hospitals or (2) self-referral. For method 1, approximately 6 hospitals and cancer centers (metropolitan and regional) across several states of Australia will be selected. The specific selection of institutions will be undertaken by the Cancer Symptoms Trials (CST) group (a national cooperative trials group) as the trial coordinating center. The CST will undertake a feasibility assessment process to ensure institutions are selected on their ability to demonstrate that they have the internal resources and patient population to refer sufficient dyads to achieve the site-specific sample size and broad coverage of services across Australia. As part of the CST site selection process, participating institutions will identify multidisciplinary staff in relevant departments to undertake an initial eligibility screen. The study will be verbally explained to eligible patients who receive a brief written project summary. With their permission, the contact details of patients interested in the study will be submitted to the project website by the relevant referring clinician. Thereafter the patient will be contacted by a research officer who will undertake the screening and consenting process incorporating obtaining permission to contact the patient’s primary family caregiver to seek their interest in participating, then scheduling a video meeting with the dyad which will also serve as a further check of their capacity to engage in an internet intervention. If all inclusion criteria are met, electronic consent via standard ethical requirements will be undertaken.

For the self-referral recruitment method, patients who have been made aware of the project (but have not been officially screened by a clinician) may self-refer via a form on the project website. These patients will be made aware via consumer, caregiver, cancer advocacy groups, or social media advertisements. A research officer will then review the self-referral form and liaise directly with the patient to advise if they meet the criteria and if so, recruiting and consent procedures will be undertaken as per method 1. There will be a second videoconference arranged (after consent and subsequent randomization) to explain to the dyad which group they have been allocated to and brief orientation to the FOCUSau platform and the associated data collection methods.

#### Allocation and Blinding

Following informed consent and completion of baseline measures, block randomization will occur via an embedded randomizer within the FOCUSau platform to create the randomization table based on blocks of multiplicators of 2 and blocks of 4, 6, and 8. The block randomization method is designed to randomize subjects into groups to ensure a balance in sample size across groups over time. A research officer will initiate the randomization process which will involve logging into the FOCUSau platform, entering relevant participant details and then block randomization will occur. Due to the nature of the intervention, the patient-caregiver dyads cannot be blinded to allocation. Data collection will occur electronically and the research team will remain blind to which trial arm the dyads were randomized until the end of the final data-collection point. The first 10 consented dyads who are allocated to the intervention group will additionally be invited to participate in the final step of the aforementioned intervention adaptation process and be provided with a specific (additional) participant information sheet and consent form.

#### Data Collection: Primary and Secondary Psychosocial Outcomes

Patient-caregiver dyad data will be collected at 3 time points—baseline (T_0_) completed prerandomization, first follow-up (T_1_) at 12 weeks post baseline, and second follow-up (T_2_) at 24 weeks post baseline. All data will be completed by the dyads (regardless of allocation) using the FOCUSau software platform, whereby they will complete a suite of internet-based questionnaires. The platform enables the application of data validation rules to ensure that required data are entered or that an annotation is provided to explain any missing data. The FOCUSau platform automatically generates a unique ID number used by the dyad to log into the platform. Data collection will be completed separately by the patient and caregiver (regardless of allocation). The data collection points, outcomes, and measures have been selected in accordance with those used in the aforementioned EU trial to enhance international comparison and potential generalizability [39].

The list of variables and associated measures to be completed by the dyad are summarized in Table 1 and are as follows, for the primary outcomes—emotional well-being (10 items describing emotional functioning from the European Organization for Research and Treatment of Cancer (EORTC) The European Organisation for Research and Treatment of Cancer Core Quality of Life Questionnaire (QLQ-C30) [55,56] and self-efficacy (The Lewis’ Cancer self-efficacy scale [57]); for the secondary outcomes—QoL of patients—EORTC QLQ-palliative care [58], 2 social functioning items (#26, 27) plus 1 item about overall health (#29) from the EORTC QLQ-C30, social well-being scale from Functional Assessment of Cancer Therapy-General (FACT-G)—and family caregivers (The Caregiver Quality of Life Index-Cancer [59]), appraisal of illness (Benefits of illness scale [60]), coping (Brief cope [61]), and dyad communication (Ways of giving support questionnaire [62] plus 1 item from the Dyadic Coping Inventory).

In addition, we will administer a sociodemographic questionnaire at baseline (T_0_) and a modified version of the Eastern Cooperative Oncology Group (ECOG) Performance Status Assessment [63] (response option 5 “death” removed as not relevant for this study).

**Table 1:** Trial data collection measures.

Objective and outcome or variable			Instrument	Timing		
				T_0_: Before randomization	T_1_:T_0_ + 12 weeks	T_2_: T_0_ + 24 weeks
**Outcome measures used for the primary outcomes**						
	**Psychosocial effectiveness**					
		Emotional well-being	For patients: EORTC^a^ QLQ-C30^b^ emotional function items (10 items) For caregivers: EORTC QLQ-C30 item emotional function scale (10 items)	✓	✓	✓
		Self-efficacy	For patients and caregivers: the Lewis’ Cancer self-efficacy scale (17 items)	✓	✓	✓
**Outcome measures used for the secondary outcomes**						
	**Psychosocial effectiveness**					
		Quality of life	For patients: EORTC QLQ-C15-PAL (23 items) + 2 social function items + 2 items about overall health from EORTC QLQ-C30; social well-being scale from the FACT-G^c^ (6 items) For caregivers: the Caregiver Quality of Life Index-Cancer (CQOLC; 35 items)	✓	✓	✓
		Appraisal of illness	For patients and caregivers: benefits of illness scale (5 items)	✓	✓	✓
		Coping	For patients and caregivers: a shortened version of Brief Cope (20 items)	✓	✓	✓
		Communication	For patients and caregivers: the “Active engagement scale” (5 items) from the “Ways of giving support questionnaire.” Three scales (10 items) from the “Dyadic Coping Inventory”: “Stress communication by oneself,” “Stress communication by partner,” and “Evaluation of dyadic coping”	✓	✓	✓
		Level of functioning	For patients (4 items): modified version of Eastern Cooperative Oncology Group (ECOG) Performance Status Assessment (item 5 “death” removed)	✓	✓	✓
	**Cost-effectiveness**					
		Health economic measures	For patients (23 items) 17 and for caregivers (14 items): client Services Receipt Inventory (CSRI) including use of hospital versus in-patient palliative EoL^d^ care and the use of informal care or services provided outside of the health care system (to be completed by caregivers) and modified to reduce overlap with Medicare data	✓	✓	✓
		Primary outcome expressed as cost per participant with a meaningful change in well-being or self-efficacy for the intervention compared with control	For patients and caregivers: EQ-5D and EQ-5D-5L (5 items)	✓	✓	✓
		Secondary outcomes expressed as cost per quality adjusted life year for the intervention compared with control	For patients and caregivers: Services Australia data—use of outpatient medical and pharmaceutical services	✓	✓	✓
	**Implementability**					
		Acceptability	For patients and for caregivers (intervention group): Theoretical Framework of Acceptability (TFA) survey (9 items)—perceptions based on info provided and actual after completion	✓	✓	✓
		Acceptability	For patients and for caregivers (control group) 1: Theoretical Framework of Acceptability (TFA) survey (9 items) perceptions based on info provided only	✓		
		Acceptability	For patients and for caregivers (intervention group only): qualitative (interviews) to explore acceptability and barriers and enablers to uptake of FOCUSau by dyads, the latter informed by the Theoretical Domains Framework of behavior		✓	
		Satisfaction with intervention	For patients and for caregivers (intervention group): FOCUS items asking about experience and satisfaction with the intervention (intervention group only) For patients (12 items) For caregivers (12 items)		✓	✓
		Uptake	Study log: recruitment data including reasons for accepting or declining participation, sociodemographic profile of invited dyads, and capacity to reach vulnerable populations (with lower socioeconomic status, cultural minorities, and from rural or remote locations)	✓	✓	✓
		Intervention fidelity	Study log: communication between trial managers and participants, whether for technical problem-solving or for other reasons. Aspects of adherence (intervention as received) are built into the FOCUSau intervention. Completion rates of individual FOCUSau sessions	✓	✓	✓
		Technical feasibility	Interviews or focus groups with health IT experts: data and infrastructure standards adherence, system architecture for interoperability or integration with electronic patient records, and extensibility and scalability potential	✓	✓	✓
	**Other characteristics**					
		Sociodemographic characteristics	For patients (14 items) and for caregivers (15 items): sex, age, relationship status, living situation, children, educational level, employment status, income, financial difficulties, medical insurance, ethnicity, and dyad’s relationship	✓		
		Patient level of functioning	For patients (1 item): modified version of Eastern Cooperative Oncology Group (ECOG) Performance Status Assessment (response option 5 “death” removed as not relevant)	✓	✓	✓

^a^EORTC: European Organization for Research and Treatment of Cancer.

^b^QLQ-C30: The European Organisation for Research and Treatment of Cancer Core Quality of Life Questionnaire.

^c^FACT-G: Functional Assessment of Cancer Therapy-General.

^d^EoL: end of life.

#### Data Collection: Health Services Use

The impact on health care costs of FOCUSau will be determined by analyzing patterns and costs of health care use, including the costs of administering the intervention. Information will be collected on the time required to administer FOCUSau (per patient and overall). The impact of FOCUSau and usual care on health care services’ use will be assessed by consenting participants for access to their Medicare (Australian Government health services) data (to obtain information on the use of outpatient medical and pharmaceutical services) and using the Client Services Receipt Inventory (CSRI) [64]; modified to reduce overlap with Medicare data. The CSRI will also allow us to explore the use of hospital versus inpatient palliative EoL care, and the use of informal care or services provided outside of the health care system (to be completed by caregivers).

#### Data Collection: Implementability

Table 1 also outlines data to be collected to measure factors related to implementability of the intervention. This will include piloting and administering a new acceptability questionnaire [44]. Interview topic guides (1 for patients; a parallel guide for caregivers) will be informed by the Theoretical Domains Framework (TDF) of behavior change [65].

#### Sample Size

A predetermined strict fixed sequence (FS) procedure defines prospectively hierarchical ordering of the primary end points; (1) emotional well-being and (2) self-efficacy. Testing of null hypotheses proceeds according to their hierarchical order; that is, H(1)0 is tested first at a significance level of 5%, and if H(1)0 is rejected then H(2)0 is tested at the same significance level, otherwise H(2)0 is not tested at all. The strict FS approach has the highest power for testing the first hypothesis (outcome: emotional well-being) compared to the other methods, as it does not save any portion of α for testing the later hypothesis. The reference mean value from EORTC for all patients with cancer, stage III-IV is 71.5 (SD 23.8). To maintain rigorous control over type I errors due to multiple comparisons, the α level is set at .025 instead of the more common .05. This adjustment accounts for the multiple comparisons required in the study, including comparisons between a control group and 2 participant groups (patients and caregivers). We set the statistical power at 0.80. The expected difference between the control group and the intervention arm in the primary outcomes is SD 0.375 at T_1_ (12 weeks). With these parameters, 173 dyads are needed in each arm (ie, 346 dyads in total). Anticipating a maximum 80% retention rate at T_1_ (US FOCUS retention was 86% [38]) we will require approximately 433 dyads to be recruited. An enrollment rate of 55% of those eligible was achieved in a prior digital health FOCUS study from 2014 [38]; however, we anticipate this will be higher for FOCUSaus (estimating 70%) given the internet is much more widely available now and our digital recruitment approach, meaning that we will need to identify approximately 618 dyads who meet eligibility criteria. Evidence also suggests that recruitment rates can increase when a digital health intervention is offered [18]. To meet these targets the CTS has calculated that approximately 6 referral sites will be required complemented by our aforementioned self-referral strategy.

#### Data Analysis

The effectiveness of FOCUSau will be compared with the standard care (control group) for each participant population (patients or caregivers). Our hypotheses will be tested using a mixed model (per participant population) with the T_1_ measurement values for emotional well-being and self-efficacy as primary outcomes using a significance level of α=.025. These mixed models will be implemented using SPSS (IBM Corp) for Windows (version 27.0; Microsoft Corp) and R (R Core Team) with the recruitment centers treated as a random effect and the randomization groups as predictor variables. As per the FS procedure, the null hypotheses of the second primary end point (self-efficacy) will only be tested if a significant result is found for the first primary end point (emotional well-being). Additionally, we will incorporate other factors identified in the literature as potentially predictive by including them as covariates in the mixed models. We will perform analyses on both “intention-to-treat” and per-protocol principles. To interpret the magnitude of the effects for the different outcomes, we will estimate effect sizes (Cohen *d*). Our analysis will encompass all primary and secondary outcomes. Primary end points, including emotional well-being and self-efficacy measured at T_1_, will be analyzed first. Following that, secondary end points, comprising outcomes measured at T_1_ that are not primary end points, as well as all outcomes measured at T_2_ (occurring 24 weeks from T_0_), will be assessed. This approach allows for a comprehensive evaluation, including the examination of longer-term effects.

The robustness and validity of the results will be explored using sensitivity analyses by varying the parameter inputs (including sensitivity to the use of values for missing observations). The analysis will be conducted within trial period; the potential to extrapolate results over the longer term will be assessed based on the proportion of patients alive at the end of follow-up.

For the cost-effectiveness analysis, costs will be reported as the mean costs of care per dyad in each arm of the study. Costs applied to health care service use will be as per Australian standard fees (eg, via the Medicare Benefits Schedule). If a difference in outcomes is observed, as hypothesized, the incremental cost-effectiveness of FOCUSau compared with control will be estimated in terms of the (1) cost per additional patient with a meaningful improvement in emotional well-being (as assessed using the EORTC QLQ-C30 emotional well-being scale) and separately, and (2) cost per additional caregiver with a meaningful change in self-efficacy (as assessed using the Lewis’ Cancer self-efficacy scale). The base case analysis of cost-effectiveness will be conducted from a health care system perspective. Subsequent sensitivity analyses will modify the assessment of costs to adopt a societal perspective to capture the impact of informal care costs, as well as test the robustness of the analysis results to variations in other parameter inputs.

Missing data for costs and outcomes will be described and summarized. Where missing data can be regarded as missing at random, likelihood (interpolation) methods will be used for the analysis of those data as appropriate.

### Stage 3: Acceptability, Feasibility, and Scalability of FOCUSau (Objective 4)

Our approach is underpinned by a conceptual framework that highlights the link between acceptability, fidelity and feasibility, and potential implementability [66]. An investigation of fidelity will also inform internal validity and thus our interpretation of the effectiveness findings. If prospective acceptability, uptake and engagement with the intervention are moderate (based on conventional cutoff scores of 50%) [67,68], then FOCUSau is feasible to deliver in other settings; if participants receive and comprehend FOCUSau as designed (ie, fidelity is high), then the effectiveness findings will represent a valid test of the intervention; if participant retention and retrospective acceptability are high, then FOCUSau is likely to be maintainable; and if FOCUSau is cost-effective and additional workforce requirements are minimal, then the intervention is likely to be scalable. Acceptability is “the extent to which people receiving FOCUSau consider it to be appropriate, based on anticipated (prospective) or experienced (retrospective) cognitive and emotional responses to the intervention” [69]. Informed by a new and influential theoretical framework of acceptability [69], we will pilot test a questionnaire [44] to be administered at baseline and follow-up (T_0_ and T_2_). As acceptability is ideally assessed both quantitatively and qualitatively [36], a diverse subsample of participants (~30 dyads) will be recruited from the intervention group and be interviewed via videoconference immediately following the completion of FOCUSau (T_1_), to gain a greater insight into intervention acceptability, including potential for further enhancements. As for the uptake, we will document the recruitment process, including reasons for accepting or declining participation and examine the sociodemographic profile of dyads invited to participate in the trial, capturing our capacity to reach relevant populations (with lower socioeconomic status, cultural minorities, and from rural or remote locations). We will also use the aforementioned T_1_ interviews to explore barriers and enablers to uptake by dyads. Interview topic guides (1 for patients; a parallel guide for caregivers) will be informed by the TDF of behavior change [65]. Intervention fidelity encompasses whether the intervention is delivered and received as planned [70]. We will document communication between trial managers and participants, whether for technical problem-solving or for other reasons, to assess whether components have been added to FOCUSau as designed. Aspects of adherence (intervention as received) are built into the FOCUSau internet module and include a readily accessible “help” function. Dyads who experience problems with onboarding for the FOCUSau sessions will receive help from the trial manager via phone; they will confirm their presence and indicate whether they are using the computer or phone; dyads who exit an FOCUSau session before completion will be sent a reminder email or text message. Completion rates of individual FOCUSau sessions will be monitored.

The audiotaped semistructured interviews with dyads to assess FOCUSau acceptability will be transcribed verbatim and thematically analyzed using the Framework method [46]. To ensure methodological rigor, the analysis will follow (1) a step-by-step guide outlined by Gale et al [71] and (2) criteria for trustworthiness [72]. The deductive analysis will be informed by the theoretical framework of acceptability (component constructs—affective attitude, burden, ethicality, intervention coherence, opportunity costs, perceived effectiveness, and self-efficacy) ascertaining whether particular elements of FOCUSau were experienced as positive or negative and will include themes from previous intervention evaluations. Coding of established themes and subthemes will be computer-assisted using NVivo software (Lumivero). Statistical descriptions of the quantitative data from the intervention checklist and routine monitoring will be used to describe fidelity, using conventional cutoff points for acceptable intervention adherence.

For technical aspects of implementability we will consider key factors in the scalability, that is “the ability of a health intervention shown to be efficacious on a small scale or under controlled conditions, to be expanded under real-world conditions to reach a greater proportion of the eligible population, while retaining effectiveness” [73]. This study will follow the previous stages and will be the subject of additional human research ethics approval. It will use health IT expert consensus methods, to determine the technical feasibility of implementing and operating the FOCUSau program as an ongoing service at each site that is participating in the clinical trial, as well as at other potential hosting sites for the program that are identified during the clinical trial. The assessment of this aspect of implementability [66] will entail either semistructured interviews or focus groups with health IT stakeholders who may include trial managers, software proprietors, digital health specialists in health service management and governance roles, and experts in the deployment of software in routine clinical care. The interview or focus group schedule will use two probes which are (1) a technical specification of the software as it has been adapted for use in the trial, based on work done by the proprietor with the research computing services group and (2) a summary of the intervention’s clinical and socioeconomic value proposition, based on preliminary findings of project studies up to this point. The interview or focus group schedule will collect and thematically analyze data, based on stakeholders’ knowledge of current local, national and international best practice approaches to digital health implementation planning [74,75], including the Australian Digital Health Agency technical standards and specifications.

### Data Management, Monitoring, and Risk Management

CST has a suite of standard operating procedures that will be used in support for the management of study data including electronic data handling, case report forms, archiving of research materials, and record destruction. Referral and recruiting reviews will be undertaken via monthly trial management video calls and ongoing oversight by the lead investigator. Monitoring will be conducted remotely, including data quality, protocol deviations or violations, adverse event reporting, participant consent, and eligibility. We will also monitor recruitment status and target achievement, review any adverse events or serious adverse events (suspected or actual), and review participant withdrawals and mortality.

### Ethical Considerations

This research protocol was approved by the Human Research Ethics Committee of St Vincents Hospital Melbourne (ERM ID 84479, SAGE Project ID 2022/PID06577, and SVHM Local Ref ID 262/22). FOCUSau is noninvasive with no known risk of protocol-related injury. As a psychoeducational intervention focused on the provision of information we anticipate the risk of any adverse events to be low. Nevertheless, information is available in FOCUSau about where participants may access additional medical and psychosocial support. Our project team is experienced in leading complex psychosocial interventions involving the recruitment of patients with advanced disease and their family caregivers. If necessary, any adverse events will be reported to the CTS Ethical and Data Monitoring Committee. Due to the nature of the study population, some deaths due to advanced disease progress are expected. A protocol for liaising with the caregiver of the deceased has been developed for these situations. This includes advising caregivers allocated to the intervention arm that they will not be required to continue with the study.

## Results

This study was funded in March 2022 and recruitment commenced in July 2024.

## Discussion

### Anticipated Principal Findings

Australia’s capacity to meet its national palliative care standards has been challenged [10]. This project will examine the clinical and health economic impacts of FOCUSau and gauge its potential for maintainability. If shown to be effective, this intervention will improve the emotional well-being of patients with advanced cancer and their family caregivers, regardless of their location or current level of health care support. Empirical findings as described above will inform an implementation and maintainability strategy. The strategy will describe a pathway and recommended steps for longer-term systematic delivery of FOCUSau across Australia, linking where possible with existing nationally supported cancer and palliative care programs.

### Comparison With Prior Research

Historically, much of the psychological and psychoeducational palliative care intervention research has been delivered in person focusing on either the patient or their family caregivers [21,23-25]. However, trials of dyadic interventions, which focus on the patient and family caregiver together, have shown favorable outcomes for both parties [18,20,21]. Furthermore, digital health interventions offer an innovative modality and may provide considerable resource advantages to in-person interventions [26,27]. Hence, our trial will advance knowledge in these areas.

### Limitations

We acknowledge some of the limitations associated with our control arm, including that control participants will not have access to FOCUSau after the final data collection point. Unfortunately, we do not have the funding and resources required to support this. We will, however, report on the number of participants who withdraw because they have not been allocated to the intervention arm.

### Conclusions

Advanced cancer is a “family affair” significantly affecting the well-being of the person with cancer and their family [1]. We seek to determine the effectiveness and maintainability of a digital health intervention (FOCUSau) aimed at improving the well-being of patients with advanced cancer and their primary family caregivers.

## Abbreviations

CSRI: Client Services Receipt Inventory
CST: Cancer Symptoms Trials
ECOG: Eastern Cooperative Oncology Group
EoL: end of life
EORTC: European Organization for Research and Treatment of Cancer
EU: European Union
FACT-G: Functional Assessment of Cancer Therapy-General
FOCUS: Family, Outlook, Communication, Uncertainty, Symptom management
FOCUSau: Family, Outlook, Communication, Uncertainty, Symptom management in the Australian context
FS: fixed sequence
iFOCUS: digital Family, Outlook, Communication, Uncertainty, Symptom management
QLQ-C30: The European Organisation for Research and Treatment of Cancer Core Quality of Life Questionnaire
QoL: quality of life
REDCap: Research Electronic Data Capture
TDF: Theoretical Domains Framework

## Data Availability

The data that support this study will be shared upon reasonable request to the corresponding author.
